# Effects of Torrefaction Pretreatment on the Structural Features and Combustion Characteristics of Biomass-Based Fuel

**DOI:** 10.3390/molecules28124732

**Published:** 2023-06-13

**Authors:** Xu Yang, Yaying Zhao, Lei Zhang, Zhuozhi Wang, Zhong Zhao, Wenkun Zhu, Jiao Ma, Boxiong Shen

**Affiliations:** 1 School of Chemical Engineering and Technology, Hebei University of Technology, Tianjin 300401, China; 202231304072@stu.hebut.edu.cn (X.Y.); 2023909@hebut.edu.cn (Z.Z.); majiao2022@hebut.edu.cn (J.M.); 2 School of Energy and Power Engineering, Northeast Electric Power University, Jilin 132012, China; 12b902035@hit.edu.cn; 3 School of Energy Science and Engineering, Harbin Institute of Technology, Harbin 150001, China; 18b902027@stu.hit.edu.cn (L.Z.); 2019919@hebut.edu.cn (W.Z.)

**Keywords:** torrefaction, biomass, structural feature, combustion kinetics, reactivity

## Abstract

Wheat straw, a typical agricultural solid waste, was employed to clarify the effects of torrefaction on the structural features and combustion reactivity of biomass. Two typical torrefaction temperatures (543 K and 573 K), four atmospheres (argon, 6 vol.% O_2_, dry flue gas and raw flue gas) were selected. The elemental distribution, compositional variation, surface physicochemical structure and combustion reactivity of each sample were identified using elemental analysis, XPS, N_2_ adsorption, TGA and FOW methods. Oxidative torrefaction tended to optimize the fuel quality of biomass effectively, and the enhancement of torrefaction severity improved the fuel quality of wheat straw. The O_2_, CO_2_ and H_2_O in flue gas could synergistically enhance the desorption of hydrophilic structures during oxidative torrefaction process, especially at high temperatures. Meanwhile, the variations in microstructure of wheat straw promoted the conversion of N-A into edge nitrogen structures (N-5 and N-6), especially N-5, which is a precursor of HCN. Additionally, mild surface oxidation tended to promote the generation of some new oxygen-containing functionalities with high reactivity on the surface of wheat straw particles after undergoing oxidative torrefaction pretreatment. Due to the removal of hemicellulose and cellulose from wheat straw particles and the generation of new functional groups on the particle surfaces, the ignition temperature of each torrefied sample expressed an increasing tendency, while the Ea clearly decreased. According to the results obtained from this research, it could be concluded that torrefaction conducted in a raw flue gas atmosphere at 573 K would improve the fuel quality and reactivity of wheat straw most significantly.

## 1. Introduction

The extensive utilization of fossil fuels in energy generation and industrial production creates serious environmental problems, such as photochemical smoke, acid rain, and especially massive emission of the greenhouse gas (GHG) CO_2_ [1]. Nowadays, it is generally believed that CO_2_ is responsible for global warming, and the imbalance of the global climate has received ever-growing attention [2,3]. Due to the carbon neutral and seasonal characteristics of agricultural biomass, the substitution of fossil fuels for agricultural by-products seems to be an appropriate method for reducing the emission of greenhouse gases during traditional power generation processes. Pyrolysis, direct combustion and gasification are the primary methods used to gain energy from biomass-based fuels [4]. During the direct combustion process, biomass reacts with adequate oxygen, accompanied by rapid energy release. However, the utilization of biomass for energy generation via direct combustion still suffers from low grindability, strong spontaneous combustion tendency, low energy density and a strong hydrophilic nature [5,6]. Previous researchers have stated that torrefied biomass generally has a better combustion performance [7]. In an attempt to improve the fuel quality of raw biomass effectively, torrefaction has been regarded as an efficient pretreatment method [8].

Torrefaction is generally performed in the temperatures range from 473 K to 573 K in inert atmosphere, and decomposition of partial hemicellulose and cellulose normally occurs during the torrefaction process [9]. During the process of upgradation conducted at relatively low temperatures (473–508 K), the weight loss of biomass mainly results from the removal of moisture content and thermal degradation of partial hemicellulose content [10]. When torrefaction temperature exceeds 508 K, the desorption of hemicellulose and cellulose tends to become apparent, along with the release of light volatiles and oxygen-containing gaseous products. With regard to severe upgradation pretreatment (548–573 K), almost all the hemicellulose and cellulose content is removed from the particles, and lignin becomes the dominant component of the torrefied sample [11]. Hemicellulose is a branched mixture containing some polymerized monosaccharides, and its chemical formula is (C_5_H_8_O_4_)_m_ [12]. Cellulose is a linear homopolysaccharide consisting of β-D-glucopyranose fragments linked together via glycosidic bonds containing some amorphous and crystalline structures; its chemical formula is (C_6_H_10_O_5_)_m_ [13,14]. Lignin is a highly branched, three-dimensional and polyphenolic material, containing an irregular array of variously bonded “methoxy-” and “hydroxyl-” substituted phenylpropane units; the chemical formula of lignin is [C_9_H_10_O_3_ (OCH_3_)_0.9–1.7_]_m_ [15]. Obviously, massive oxygen atoms exist in the structures of hemicellulose and cellulose, leading to higher atomic ratios of O/C (0.80 and 0.83, respectively) than that of lignin (0.39–0.44). Furthermore, the combustion characteristic and reactivity of torrefied biomass has been found to be similar to that of lignite or bituminous [16]. Therefore, increasing lignin content via torrefaction at the expense of hemicellulose and cellulose seems to be beneficial to increasing the energy density of biomass-based fuels.

Flue gas torrefaction is a special type of oxidative torrefaction, which has been regarded as a new and promising biomass upgradation method [17,18]. Due to the recycling of waste heat from combustion flue gas, the energy demand for biomass upgradation can be reduced significantly [19]. Moreover, the oxidizing agents (mainly O_2_, CO_2_ and H_2_O) in the flue gas react with several organic components in biomass particles and release additional heat via a self-sustained thermal process, reducing the energy consumption of torrefaction [20]. Due to the synergistic effects of devolatilization, surface oxidation and gasification, the reaction rate for oxidative torrefaction is normally faster than that of inert torrefaction, which can apparently shorten the duration and lower the operation temperature required to achieve an identical upgradation target [21]. Increasing torrefaction severity could significantly affect the quality and composition of the solid product; products obtained from oxidative upgradation generally expressed a higher heating value accompanied by lower mass yield [22]. Compared to inert torrefaction, oxidative torrefaction (especially flue gas torrefaction) has more advantages and greater application potential, and seems to be more feasible in commercial and industrial applications.

During oxidative torrefaction processes, the attachment of oxidizing agents and the desorption of oxygen-containing functionalities tended to occur simultaneously, resulting in substantial variations in the physicochemical structure and compositional distribution of biomass [23]. Due to the synergistic effects of devolatilization and surface oxidation, the efficiency for deoxygenation, dehydrogenation and carbonization during oxidative upgradation processes become more significant. Surface oxidation is more likely to enlarge the porosity of particles through the collapse of macrospores and the removal of active organic components, accompanied by selected consumption of hydrophilic groups [24]. Therefore, upgraded biomass derived from oxidative torrefaction normally has a larger surface area, stronger reactivity, higher thermal stability and better palletization [25]. However, few studies have clarified the correlation between combustion kinetics, surface physicochemical properties and fuel quality of torrefied biomass derived from flue gas torrefaction, and only limited information is available.

Aiming to achieve high-efficiency utilization of waste heat and oxidizing components in combustion flue gas, flue gas was employed for the upgradation of agricultural solid waste in this study. The fuel quality, physicochemical feature and combustion reactivity of torrefied wheat straw derived from various torrefaction conditions were investigated systemically. Then, the distribution of typical elements and fiber components was identified via elemental analysis and fiber content determination. Moreover, the evolution characteristics of typical functional groups on the particle surfaces were studied through XPS spectra. The effects of torrefaction severity on the pore structural features of wheat straw particles was also identified through the N_2_ adsorption method. By means of the utilization of the TGA method, typical combustion characteristic parameters and combustion kinetics of each torrefied sample could be identified. There might be some differences between the experimental conditions of this study and real torrefaction conditions, but the results obtained from this work will provide some fundamental information and theoretical basis for optimizing the existing operation condition of biomass upgradation at an industrial scale.

## 2. Results and Discussion

### 2.1. Clarification of Componential Variation

#### 2.1.1. Characteristics of Change in Organic Contents during Torrefaction Process

**Figure 1:**
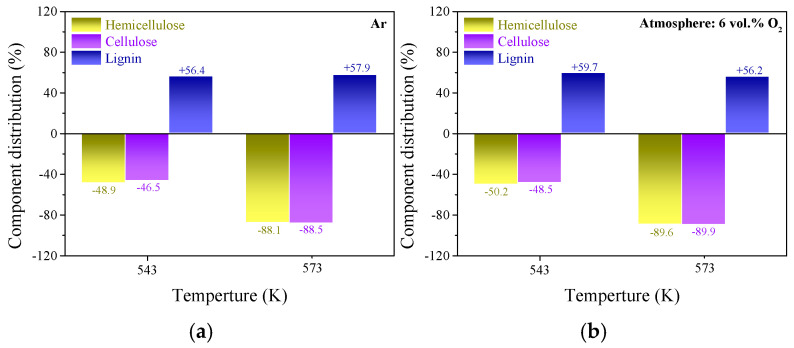

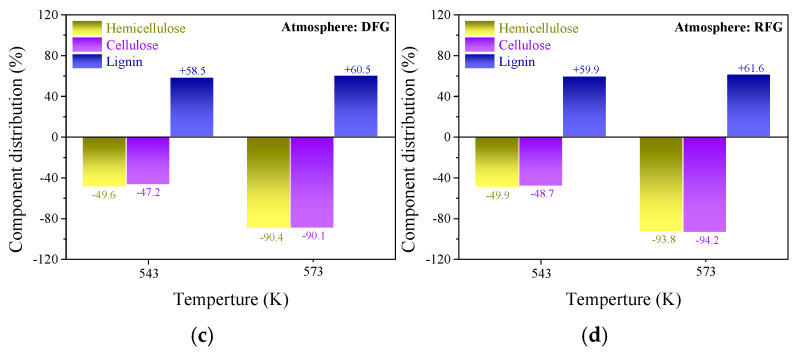
Variations in the content of hemicellulose, cellulose and lignin in the torrefied samples. (**a**–**d**) the specific values for torrefied samples derived from inert, oxygen deficient, DFG and RFG torrefaction, respectively.

Hemicellulose, cellulose and lignin are the primary organic contents of lignocellulosic biomass [26]. The fiber content analysis illustrated that hemicellulose and cellulose were the main components in raw wheat straw particles, and the specific contents of hemicellulose, cellulose and lignin in raw wheat straw were approximately 19.24%, 44.82% and 26.33%, respectively. Due to the high proportion of active components (hemicellulose and cellulose), wheat straw generally expressed the characteristics of low thermal stability and high reactivity, implying a great risk of spontaneous combustion and degradation during storage and transportation. Torrefaction pretreatment has been proven to be an effective method for reducing the spontaneous combustion tendency, improving the fuel quality and hydrophobicity of biomass [3,4]. Unlike lignin, hemicellulose and cellulose contained several polysaccharides with a lower degree of polymerization, a larger relative amount of available functional groups and higher O/C atomic ratio values (approximately 0.82 for hemicellulose and 0.83 for cellulose) [19]. After undergoing torrefaction pretreatments, the specific content for each fiber component in the upgraded samples showed a significant difference; the results are summarized in Figure 1.

After undergoing torrefaction pretreatment, the content of lignin for each sample increased (by 56.2–61.6%) at the expense of hemicellulose and cellulose. This phenomenon implied that the emission of light volatiles and oxygen-containing gaseous species derived from the decomposition of hemicellulose and cellulose was more likely to be accelerated by the participation of oxidizing agents during the torrefaction process, which was consistent with the results obtained by previous researchers [27]. The increase in lignin content reached the maximum value of 61.6% at the temperature of 573 K in the raw flue gas atmosphere, indicating that the energy yield and heating value of the upgraded sample pretreated in these conditions ought to be larger than for the other torrefaction conditions. According to the results in Figure 1, it can be assumed that the addition of CO_2_ and H_2_O was beneficial to strengthening the wheat straw torrefaction degree, especially at high temperatures. Massive polysaccharide components and functionalities with low thermal stability (mainly hydroxyl and carboxyl) were removed due to the synergistic activation of multiple oxidants during the torrefaction process [28], so the thermal stability and hydrophobicity of the upgraded sample were increased. These improvements in fuel quality guaranteed the safe storage and efficient utilization of wheat straw. Therefore, the utilization of raw flue gas for the torrefaction of agricultural by-products seems to be a potential approach.

#### 2.1.2. Effects of Torrefaction Conditions on Elemental and Compositional Distribution

**Figure 2:**
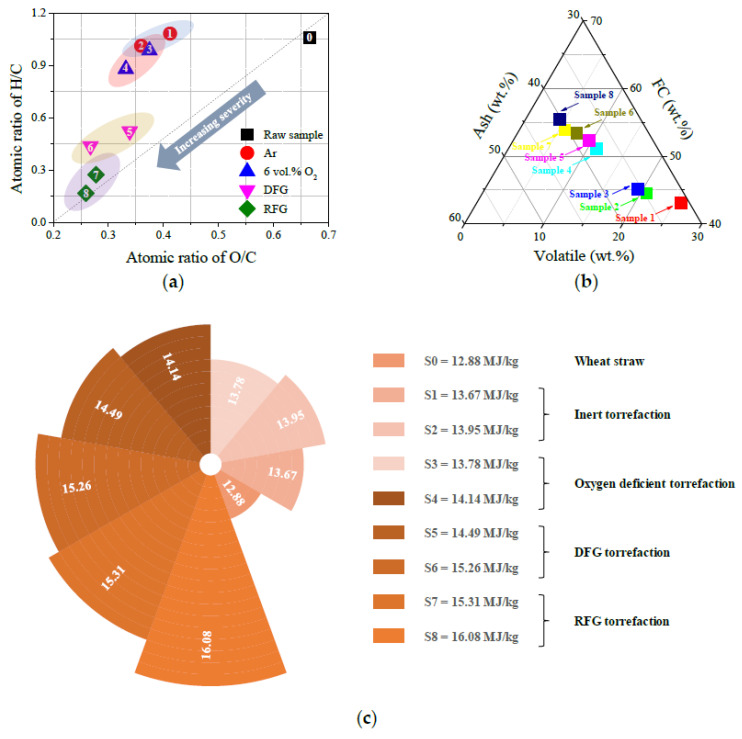
Characterization of wheat straw samples torrefied under various conditions. (**a**) Elemental distribution; (**b**) compositional distribution; (**c**) HHV values for raw and torrefied samples.

According to the results in Table 1, the relationship between upgradation condition and biomass elemental distribution was identified, and the specific results are illustrated in Figure 2a. The chemical formulae of hemicellulose, cellulose and lignin are (C_5_H_8_O_4_)_m_, (C_6_H_10_O_5_)_m_ and [C_9_H_10_O_3_ (OCH_3_)_0.9–1.7_]_m_, respectively, so the O/C atomic ratio values for hemicellulose, cellulose and lignin are approximately 0.82, 0.83 and 0.53, respectively [13,19]. The decomposition of hemicellulose accelerates the cleavage of glycosidic bonds and the desorption of O-acetyl groups, as well as methylene on side chains, accompanied by the emission of oxygen-containing gaseous species [29]. The variations in the elemental distribution for each torrefied sample derived from different upgradation processes could be attributed to the almost complete removal of hemicellulose, along with massive removal of cellulose and slight oxidation of lignin in wheat straw particles. Additionally, Silva et al. [26] and Wang et al. [27] showed that an oxidizing atmosphere was beneficial to promote the release of light volatile materials during the thermal conversion process of carbonaceous materials. This is consistent with the phenomena observed during this research; the devolatilization tends to be strengthened during oxidative torrefaction processes. Therefore, the ash content and fixed carbon content are more likely to increase at the expense of volatile content.

The results in Table 1 and Figure 2a show that increasing torrefaction temperature increases the relative amount of carbon content in upgraded wheat straw particles (49.44 wt.% → 72.69 wt.%), leading to an apparent reduction in the atomic ratio values of H/C and O/C synchronously. Meanwhile, it could be assumed that the participation of oxidizing agents in the upgradation of carbonaceous materials generally tends to promote the decomposition of reactive functional groups accompanied by the release of gaseous products through deoxygenation, decarbonization and dehydrogenation. This phenomenon is consistent with the results obtained by previous studies [27]. Thus, increasing torrefaction severity decreases the atomic ratios of H/C and O/C, which could be attributed to the polymerization between benzene ring structures and the synergistic acceleration of C(O) desorption. The upgraded sample derived from torrefaction occurred in an RFG atmosphere at 573 K located at the closest position to the bottom left in the van Krevelen diagram, reflecting that the carbon content was the highest in sample 8.

As the results summarized in Table 1 show, the relative amount of volatile and fixed carbon in raw wheat straw particles was 56.13 wt.% and 20.45 wt.%, respectively, indicating a relatively low energy density (HHV = 12.88 MJ/kg). The results in Figure 2b illustrate that torrefaction pretreatment increased the relative amount of fixed carbon at the expense of volatile-through-mild devolatilization, surface oxidation and gasification. Previous researchers have shown that more volatile materials would be released in an oxidizing atmosphere than in an inert atmosphere, especially at high temperatures [15]. The sample obtained from torrefaction performed in an RFG atmosphere (6 vol.% O_2_ + 10 vol.% CO_2_ + 5 vol.% H_2_O) at 573 K had the highest energy density and HHV value, and this assumption was consistent with the calorific value measurement results in Figure 2c. Generally, the fuel with higher volatile content tended to have higher ignition temperature reactivity. Due to the removal of high-oxygen-content components (hemicellulose and cellulose) from wheat straw particles, the HHV value (12.88 MJ/kg → 16.08 MJ/kg) and fixed carbon content (20.45 wt.% → 53.98 wt.%) for each torrefied sample increased after undergoing torrefaction pretreatment. Moreover, it has often been speculated that torrefaction pretreatment would effectively improve the thermal stability of biomass, reduce the risk of spontaneous combustion and increase the safety of storage and transportation [30]. Thus, the specific combustion characteristic parameters and kinetic parameters of the torrefied samples derived from various torrefaction conditions are analyzed using the TGA method in the following section.

### 2.2. Fuel Quality Identification

Mass yield (*Y*_M_) and energy yield (*Y*_E_) are generally regarded as two vital factors reflecting the mass loss and energy densification of biomass-based fuels derived from torrefaction pretreatment [31]. By means of the employment of these fuel quality indexes, the thermal conversion characteristics (pyrolysis, gasification and combustion) of upgraded biomass can be indicated effectively. The specific values of *Y*_M_ and *Y*_E_ for each torrefied sample were determined using Equations (1) and (2) [32]. The energy–mass co-benefit index (*EMCI*) was defined to quantify the increase in energy content for the remaining mass after undergoing torrefaction process, which was calculated with Equation (3) [33]. The subscripts “r” and “t” in these equations illustrate raw and torrefied wheat straw, respectively.

**Figure 3:**
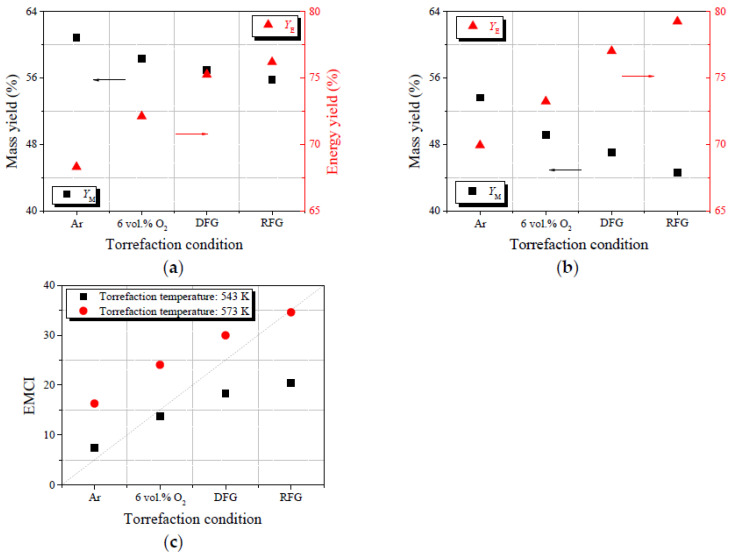
Variations in fuel quality. (**a**,**b**) Mass yield and energy yield for each sample torrefied at 543 K and 573 K; (**c**) *EMCI* values for the torrefied samples.

The calculated results of *Y*_M_, *Y*_E_ and *EMCI* for each upgraded sample were determined via Equations (1)–(3), and the specific results are summarized in Figure 3. Both rising torrefaction temperature (543 K → 573 K) and oxidizing agent volumetric fraction (Ar → RFG) tended to monotonously decrease the *Y*_M_ (60.88% → 44.66%), while increasing the *Y*_E_ (68.31 → 79.24%). This phenomenon can be attributed to the acceleration of the desorption rate for hemicellulose and cellulose resulted from torrefaction severity enhancement [31]. The removal of hemicellulose and cellulose tended to increase the carbon content in biomass particles, resulting in higher thermal stability and energy density of torrefied samples. Previous studies have stated that *EMCI* is generally employed as an index for reflecting the optimal torrefaction conditions that satisfy the requirement of upgraded biomass with high energy density and low volume, which could reduce the costs of transportation and storage [33]. The results in Figure 3c illustrate this; rising torrefaction severity increased the value of *EMCI* (7.43 → 34.58), and the conclusion was consistent with those obtained by other researchers [7]. Based on the results in Figure 3, it could be assumed that torrefaction performed in an RFG atmosphere at 573 K was more suitable for wheat straw upgradation.

### 2.3. Analysis of Surface Chemical Structure

#### 2.3.1. Effects of Reaction Condition on the Distribution of C(N)

**Figure 4:**
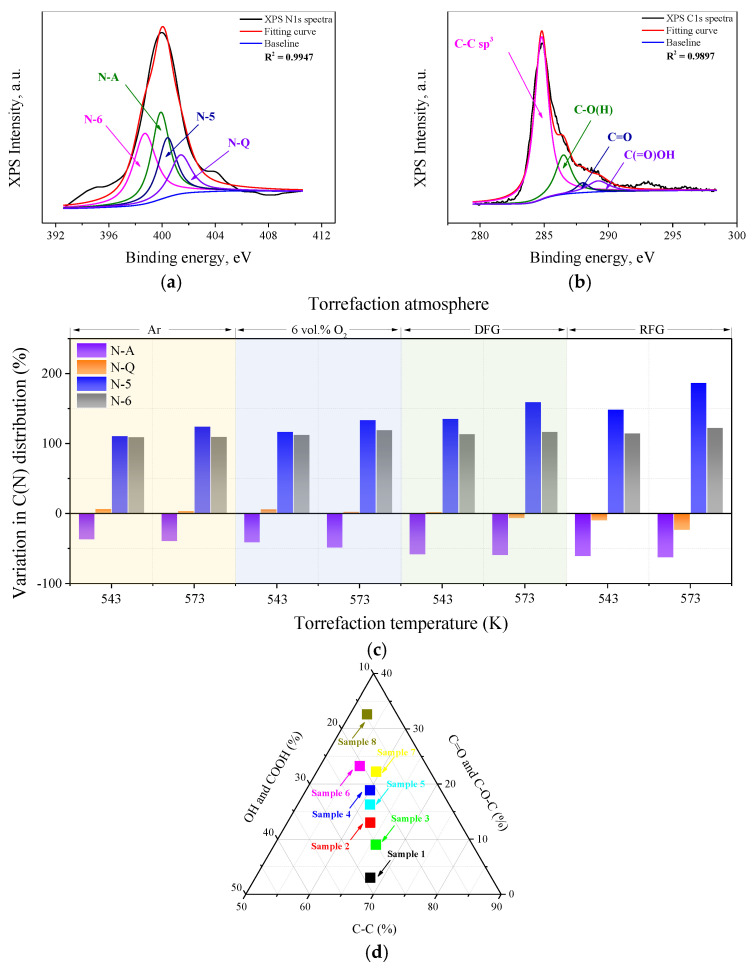
XPS deconvolution results of each upgraded sample. (**a**,**b**) deconvolution example of XPS N1s spectra and XPS C1s spectra for sample 1; (**c**,**d**) evolution characteristics of nitrogen and oxygen-containing functional groups, respectively.

XPS analysis was employed in this study to clarify the change in behavior of structural nitrogen in wheat straw particles after undergoing various torrefaction conditions. According to the specific assignment of each typical nitrogen-containing functional group, the XPS N1s spectra could be deconvoluted into four peaks (N-5, N-6, N-A and N-Q) [34]. Due to the participation of oxidizing agents in the thermal conversion process of carbonaceous materials, the microstructure and the occurrence form of typical elements was altered significantly. The relative amount of each type of nitrogen-containing functionality could be determined semi-quantitatively through the integration of each deconvoluted peak in the spectra. The distribution characteristics of structural nitrogen on the surface of the torrefied samples derived from various pretreatment conditions are summarized in Figure 4c.

As illustrated in Figure 4c, the evolution characteristics of structural nitrogen during the torrefaction process was heavily dependent on the upgradation atmosphere and temperature. N-A was normally regarded as the overwhelming form of nitrogen content in biomass, while the relative amount of inside nitrogen (N-Q) and edge nitrogen (N-5 and N-6) was very low [35]. The increase in torrefaction severity was more likely to promote the conversion of N-A into nitrogen-containing gaseous species and nitrogen-containing functional groups through thermal desorption and crosslinking reactions [34]. Due to the activation of C-N and C-C bonds by oxidizing atoms during the torrefaction process, the generation and enrichment of edge nitrogen structures (N-5 and N-6) was clear [36]. This phenomenon implied that surface oxidation (wheat straw + O_2_) and gasification (wheat straw + CO_2_ or/and H_2_O) tended to synergistically promote the conversion of nitrogen atoms initially existing as N-A and aromatic structural units (N-Q) into the edge of structural units (N-5 and N-6). Additionally, the reaction between wheat straw particles and oxidizing agents can also promote the destruction of condensed aromatic structures into small ones, effectively strengthening the reactivity of each torrefied sample at high upgradation temperatures. Thus, increasing the torrefaction temperature (543 K → 573 K) and volumetric fraction of oxidizing agent (100 vol.% Ar → 6 vol.% O_2_ + 10 vol.% CO_2_ + 6 vol.% H_2_O) seemed to increase the content of edge nitrogen (N-5 and N-6) in the upgraded wheat straw particles, especially N-5, which is generally regarded as a precursor to HCN and NH_3_.

#### 2.3.2. Effects of Reaction Condition on the Distribution of C(O)

There were four hidden peaks in the XPS C1s spectra, which were associated with C-C with sp^2^ and sp^3^ bonding (284.8–285.4 eV), carbonyls (287.8–288.2 eV), epoxides or hydroxyls (286.4–286.8 eV) and carboxyl acidic groups (288.3–289.2 eV), respectively [37]. The carboxyls and hydroxyls are hydrophilic groups which can adsorb H_2_O molecules through hydrogen bonds [10]; both carboxyl and hydroxyl ought to be removed through upgradation pretreatment to strengthen the hydrophobicity of torrefied samples. Furthermore, carbonyls are the precursors to CO, which has the potential homogeneously to reduce NO into N_2_ in oxygen-deficient atmosphere at high temperatures [16]. With the application of the deconvolution method to the XPS C1s spectra of each upgraded sample, the effects of torrefaction conditions on the evolution characteristics of oxygen-containing functional groups could be clarified semi-quantitatively. The deconvolution results for each upgraded sample are summarized in Figure 4d.

Because the upgradation of wheat straw was performed in various oxidizing atmospheres, the torrefaction process mainly consisted of devolatilization, thermal degradation, surface oxidation and gasification simultaneously. As the results illustrate in Figure 4d, increasing torrefaction temperature (543 K → 573 K) reduces the relative amount of hydrophilic groups in identical upgradation atmospheres. This phenomenon might be attributed to the low thermal stability of phenol and carboxyl. Nevertheless, with the increase in torrefaction temperature, the evolution characteristics of C(O) exhibited an opposite tendency to that of the hydrophilic groups. In oxidative torrefaction processes, the thermal degradation of hemicellulose and cellulose, which contain abundant hydrophilic structures, is normally accompanied by rapid generation of new C(O) (ether, lactone, etc.) [38]. More C(O) generally reflects more defects and surface active sites on the surface of carbonaceous materials [31]. Meanwhile, the attachment of oxidizing atoms tends to promote the conversion of originally condensed aromatic structures into small and reactive ones, and the participation of more oxidizing agents in the torrefaction process accelerates this phenomenon [10]. Additionally, the hydrogen free radicals derived from the decomposition of H_2_O molecules are more likely to penetrate into the carbon matrix of carbonaceous materials, strengthening the removal or conversion of hydrophilic groups (especially carboxyl) [36]; raw flue gas torrefaction is therefore more suitable for optimizing the fuel quality and strengthening the hydrophobicity of upgraded wheat straw synchronously. Based on the results above, it could be assumed that wheat straw upgraded in raw flue gas at 573 K tended to reach the highest reactivity and the strongest hydrophobicity.

### 2.4. Surface Physical Structure Determination

**Figure 5:**
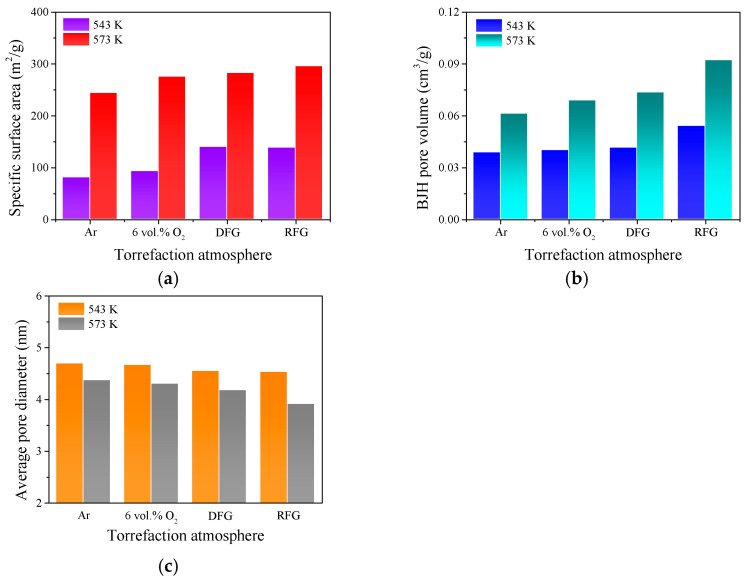
Identification of surface pore structural features of each torrefied sample. (**a**) The specific surface area; (**b**) the BJH pore volume; (**c**) the average diameter of pore structure.

With the utilization of the BET and BJH methods, the specific surface area, pore volume and pore size of each upgraded sample could be determined through N_2_ adsorption analysis. Variations in torrefaction condition tended to have significant impacts on the physical structure of wheat straw. Biomass torrefaction could be regarded as a mild devolatilization and surface oxidation process; the depolymerization and destruction of partial macromolecules such as hemicellulose and cellulose occurred simultaneously with the desorption of gaseous species and tars [27]. This phenomenon seemed to be beneficial to opening or linking some initially closed pores, enlarging the surface area of torrefied particles obviously. The specific surface area, pore volume and average pore diameter of each torrefied sample derived from different upgradation conditions are summarized in Figure 5.

Based on the N_2_ adsorption results in Figure 5, it could be assumed that torrefaction tends to promote the formation of advanced pore structures on the surface of biomass particles, especially in oxidizing atmospheres; this is consistent with previous studies [31]. Meanwhile, torrefaction temperature was the most vital factor affecting the evolution characteristics of biomass surface physical structure. As the results in Figure 5 illustrate, increasing torrefaction temperature (543 K → 573 K) tends to enlarge the specific surface area and pore volume, along with an obvious reduction in the average diameter of pore structure. For lignocellulosic biomass, cellulose micro-fibrils were normally embedded in a matrix of hemicellulose contents, and the layers of lignin tended to pack hemicellulose and cellulose densely [15]. Due to the rapid thermal degradation of hemicellulose content in the oxidizing upgradation processes, the destruction of thick-walled fibers in the particles tended to be promoted, which accelerates the rapid desorption of abundant inclusions as well as the drastic formation of tube-like pore structures. Due to the reduction in fragmentation intensity and enhancement in homogeneity resulting from increasing torrefaction severity, the average pore diameter decreased, accompanied by significant enhancement in pore volume [39]. When the torrefaction temperature remained identical, the upgraded samples obtained from raw flue gas normally had the largest surface area and pore volume, and the smallest average diameter. This phenomenon illustrates that the addition of more oxidizing agents tended to result in a rapider shrinkage or collapse of large pore structures into small ones, as well as the generation of new pores through severe volatile release and organic component (hemicellulose and cellulose) consumption, which was consistent with the results obtained by the previous studies [40,41]. Furthermore, advanced surface pore structure was conducive to promote the diffusion of oxidizing atoms to the active sites on the particle surface, strengthening the subsequent utilization of the upgraded wheat straw. Therefore, raw flue gas seemed to be the most suitable atmosphere for agriculture by-product upgradation, and the torrefaction temperature ought to remain at around 573 K.

### 2.5. TGA Investigation

#### 2.5.1. Combustion Characteristic Parameters of Each Upgraded Sample

**Figure 6:**
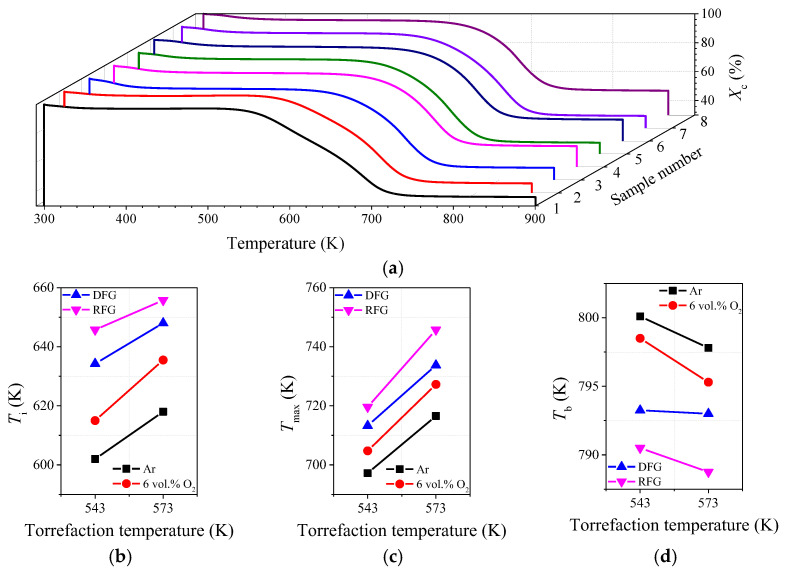
Combustion characteristic parameters for each upgraded sample. (**a**) TGA curves for each torrefied sample; (**b**–**d**) the ignition temperature, maximum weight loss temperature and burnout temperature of each sample.

TGA has been proven to be an effective method for determining the combustion characteristics of carbonaceous materials. By means of analyzing the TG and DTG curves of each torrefied sample, the specific combustion characteristic parameters such as ignition temperature (*T*_i_), the maximum weigh loss temperature (*T*_max_) and the burnout temperature (*T*_b_) could be determined through the TGA curves in Figure 6a [42], and the specific results are shown in Figure 6. At a low temperature range (about 300–400 K) of TG profile, the weight loss of each sample could be associated with the removal of moisture content re-adsorbed in upgraded particles. Generally, a peak appeared in the medium-temperature region (about 550–650 K) of the DTG curve of each sample, which could be attributed to the thermal degradation of reactive organic components with relatively low thermal stability (mainly hemicellulose and cellulose) [43]. However, the results in Figure 1 illustrate that massive hemicellulose and cellulose was removed from wheat straw particles after undergoing torrefaction pretreatments, and lignin became the dominant component in each torrefied sample. When the torrefaction temperature exceeded 650 K, the weight loss of each upgraded sample could be mainly associated with decomposition and oxidation of lignin [44]. Therefore, compositional differences would lead to different combustion characteristics of the torrefied samples.

There were massive reactive oxygen-containing functional groups with relatively low thermal stability in hemicellulose and cellulose. Increasing torrefaction severity, especially reaction temperature, lowered the relative amount of hemicellulose and cellulose, as shown in Figure 1. The results in Figure 6 show that, when the torrefaction temperature increased from 543 K to 573 K, the variations in torrefaction atmosphere had more apparent impacts on the ignition temperature (602 K → 655 K) and the maximum weight loss temperature (697 K → 746 K) for each upgraded sample. This phenomenon might be attributed to the significant compositional variations (as shown in Figure 1). Due to the cleavage of glycosidic bonds, the destruction of O-acetyl groups, the decomposition of methylene on side chains, as well as the fragmentation of structural units [29], massive hemicellulose decomposed during the torrefaction processes. Meanwhile, the oxidizing atoms were more likely to activate the chemical bonds in the structural units, leading to significant decomposition of glycosidic bonds in cellulose [7]. While lignin was composed of macromolecules and aromatic units which were thermally stable, so the desorption rate of lignin was apparently lower than that of hemicellulose and cellulose under torrefaction conditions [26]. Additionally, increasing torrefaction severity was more likely to promote polymerization between the benzene ring structures, enhancing the ignition temperature of torrefied samples remarkably (602 K → 655 K) [45]. On the other hand, the additional oxidizing agents would attach to the surface of particles generating oxygen-containing functional groups (as illustrated in Figure 4d), which increased the reactivity of torrefied wheat straw particles. Therefore, with the increase in torrefaction severity, the burnout temperature of wheat straw decreased clearly (800 K → 788 K). According to the results in Figure 6, it could also be concluded that torrefaction pretreatment for wheat straw ought to be conducted in an RFG atmosphere at 573 K to achieve the most effective optimization of combustion characteristics.

#### 2.5.2. Combustion Kinetics of Each Torrefied Sample

In this study, the conversion ratio from 0.10 to 0.50 was employed for the determination of combustion activation energies for torrefied samples at three different heating rates (5, 10, 15 K/min). The results are summarized in Table 2. The average activation energies of the torrefied samples pretreated at 543 K in inert, 6 vol.% O_2_, DFG and RFG atmosphere were found to be 139.75 kJ/mol, 134.18 kJ/mol, 138.91 kJ/mol and 130.99 kJ/mol, respectively. Meanwhile, the average activation energies of the pretreated wheat straw torrefied at 573 K in inert, 6 vol.% O_2_, DFG and RFG atmospheres were 174.89 kJ/mol, 161.40 kJ/mol, 130.99 kJ/mol and 129.30 kJ/mol, respectively. Due to the significant decomposition of hemicellulose and cellulose (as shown in Figure 1) during torrefaction processes, increasing torrefaction temperature (543 K → 573 K) generally expressed negative effects on strengthening the reactivity of torrefied samples (Ar: 139.75 kJ/mol → 174.89 kJ/mol, 6 vol.% O_2_: 134.18 kJ/mol → 161.40 kJ/mol). However, when the torrefaction pretreatments were performed in RFG and DFG atmospheres, the torrefied samples generally had lower *E*_a_ values than those obtained from inert and oxygen-deficient atmospheres (as illustrated in Table 2), especially at high torrefaction temperature (573 K). This phenomenon might be attributed to the attachment of oxidizing agents to the surface of biomass particles during torrefaction processes, generating oxygen-containing functional groups which could strengthen the reactivity of carbonaceous materials [38]. This assumption was consistent with those results illustrated in Figure 4. Moreover, the multiple oxidants could synergistically promote the conversion of some dense aromatic ring structures in biomass particles into small and reactive ones during the pretreatment process, increasing the reactivity of torrefied wheat straw [46]. According to the kinetics calculation results and the experimental results obtained above, the torrefied wheat straw derived from RFG torrefaction conducted at 573 K expressed the best fuel quality and the highest reactivity. Therefore, the optimum torrefaction condition for wheat straw is an RFG atmosphere at 573 K.

## 3. Experimental

### 3.1. Sample Preparation

Wheat straw, a typical lignocellulosic agricultural solid waste, was employed as the raw material in this work. The raw wheat straw was initially dried in an oven at 378 K for 48 h to thoroughly remove the inherent moisture, and the dried sample was subsequently grinded and sieved into a uniform size distribution (100–130 μm). The torrefaction experiments were conducted in a fixed-bed horizontal experimental system, as shown in a previous study [47]. The torrefaction experiments were performed in various atmospheres at two typical temperatures (543 K and 573 K) for 30 min. Approximately 1 g raw wheat straw sample was used for each run. The specific reaction conditions and abbreviations are illustrated in Table 3. The reaction gas flowed from the lower end of the vessel and flowed out from the upper end at the rate of 1.5 L/min; the wheat straw particles could flow with the carrier gas, presenting a fluidization state during the whole torrefaction process, effectively eliminating the interference on the experimental results of secondary reactions resulting from particle stacking.

The proximate analysis of raw and torrefied wheat straw was carried out using an Industrial Analyzer (5E-MAG6700, the Kaiyuan Instruments Co., Ltd., Changsha, China). The ultimate and heating values for each sample were determined using an Elemental Analyzer (vario MACRO Cube, ELementar, Hanau, Germany) and a Heating Value Analyzer (ZDHW-9000, Xianfeng Instrument Co., Ltd., Shijiazhuang, China). Moreover, the ash content composition of raw wheat straw was also determined according to the National Standards of PRC and the GB/T 30725-2014 [48]. The basic results for raw and torrefied samples are summarized in Table 1.

### 3.2. Fuel Quality Identification

*Y*_M_ and *Y*_E_ are two vital indexes illustrating the mass loss and energy densification improvement of biomass after undergoing torrefaction pretreatment [9]. In order to identify the torrefaction performance of each upgraded sample obtained from various upgradation processes, the *Y*_M_ and *Y*_E_ values for each torrefied sample were calculated with Equations (1) and (2), respectively. The *Y*_M_ reflects the residual amount of solid matter, while the *Y*_E_ indicates the heating energy remaining in each torrefied sample. Furthermore, the *EMCI* illustrates the increase in energy content for the remaining mass after undergoing upgradation pretreatment, as illustrated in Equation (3) [7].

(1) $$Y_{M}\;(\%,\;\mathrm{db})=\frac{{\mathrm{Weight}}_{t}}{{\mathrm{Weight}}_{r}}\times 100$$

(2) $$Y_{E}\;(\%,\;\mathrm{db})=\frac{{\mathrm{Weight}}_{t}\times {\mathrm{HHV}}_{t}}{{\mathrm{Weight}}_{r}\times {\mathrm{HHV}}_{r}}\times 100$$

*EMCI* = *Y*_E_ − *Y*_M_ (3)

### 3.3. XPS Analysis

By using an XPS furnace (PHI 5400 ESCA system, RBD Instruments, Bend, OR, USA) equipped with an Al Kα X-ray source (*hv* = 1486.6 eV), the specific distribution of typical functional groups on the surface of torrefied particles could be determined semi-quantitatively [38]. The C1’s calibration energy was 284.80 eV. The pass energy was fixed at 93.9 eV to ensure the high sensitivity of this furnace; the narrow and wide spectra were subsequently recorded with a high resolution. The pass energy the of narrow and wide scans were 22.35 and 178.95 eV, respectively. Generally, the structural features of functional groups affects the reactivity of carbonaceous materials and the emission characteristics of gaseous products. By means of deconvoluting the XPS spectrum using the PeakFig v4.12 software, the specific characteristics of change in typical functional groups during the torrefaction process could be clarified semi-quantitatively.

### 3.4. Pore Structure Determination

The pore structural characteristics of each sample were studied using a N_2_ adsorption device (ASAP 2020M, Micromeritics Instrument Corp., Norcross, GA, USA). The specific technical parameters of the furnace were as follows: (a) the specific surface area ranged from 0.0005 m^2^/g to infinity; (b) the pore size ranged from 0.35 to 500 nm and (c) the micropore resolution was 0.02 nm [49]. The adsorption test was conducted at 77 K for each run. The samples derived from various upgradation conditions were degassed in the adsorption system at 313 K to a final pressure of 1.33 × 10^−4^ Pa. Subsequently, the average pore diameter and specific surface area could be calculated through the BET and BJH methods, respectively [50].

### 3.5. TGA Test

Non-isothermal thermogravimetric analysis (SDT Q600, TA Instruments, New Castle, DE, USA) has been proven to be applicable for identifying the relationship between upgradation condition and combustion characteristics of each torrefied sample by other researchers [51]. Approximately 5 mg upgraded sample was weighed for each TGA test, and the reactor was heated from room condition to the target temperature of 873 K at a heating rate of 10 K/min in air atmosphere with a flow rate of 0.2 L/min. The weight loss curve (TGA profile) and the DTG for each upgraded sample were recorded and analyzed using the OriginPro 9.0 software. Based on the weight loss curve (TGA) and its first derivative curve (DTG) for each torrefied sample, the specific combustion characteristic parameters for each upgraded sample could be determined through parallel tests.

Furthermore, the TGA was conducted at various heating rates of 5, 10 and 15 K/min to determine the kinetic parameters of each wheat straw sample. The reaction rate expresses the conversion ratio as a function of rate constant and conversion ratio at a constant temperature [31]. The conversion ratio *a* was calculated with equation (5). The kinetic parameters pre-exponential factor (*A*) and activation energy (*E*_a_) represent the reaction complexity and the energy threshold, respectively [32]. In order to determine the values of *A* and *E*_a_ for each sample, the Arrhenius equation (6) was employed to illustrate the correlation between reaction rate and reaction temperature during the process of a heterogeneous solid-state reaction [33].

(4) $$\frac{da}{d\tau }=kf\left(a\right)$$

(5) $$a=\frac{\left(m_{0}-m_{t}\right)}{\left(m_{0}-m_{\infty }\right)}$$

(6) $$k=Ae^{-E_{a}/RT}$$

(7) $$\frac{da}{d\tau }=A\exp\left(\frac{-E_{a}}{RT}\right)f\left(a\right)$$

(8) $$\beta =\frac{dT}{d\tau }$$

(9) $$\frac{da}{dT}=\frac{A}{\beta }\exp\left(\frac{-E_{a}}{RT}\right)f\left(a\right)$$

(10) $$G\left(a\right)=\int_{T_{0}}^{T}\frac{A}{\beta }\exp\left(\frac{-E_{a}}{RT}\right)dT$$

(11) $$\ln\beta =-1.052\frac{E_{a}}{RT}+\left\{\ln\left[\frac{AE_{a}}{RG\left(a\right)}\right]-5.311\right\}$$

The model-free method Flynn–Ozawa–Wall (FOW), which is widely used for analyzing the thermal conversion kinetics of biomass-based fuels, was employed to determine the kinetic parameters of each upgraded sample. The FOW method uses Doyle’s approximation for *E*_a_ calculation. For each TGA process, the heating rate *β* remained constant, and Equation (7) was modified to Equation (9). Meanwhile, the mechanism function *G*(*a*) was expressed with respect to *a* or *T*, as shown in Equation (10). Several assumptions were made for the kinetic determination: (a) the widely accepted solid-state reaction models and orders, which were summarized by previous studies [32,52]; (b) the reaction rate at constant conversion ratio in model-free methods was assumed to be only affected by reaction temperature. By means of the utilization of the FOW method, *E*_a_ could be determined using the slope of the plots between lnβ and 1/T. Therefore, the regulation of change in *E*_a_ within the conversion range of 0.1–0.5 could also be identified.

## 4. Conclusions

Experimental investigations of surface behavior and reactivity of wheat straw derived from various torrefaction conditions were performed in this work. The fuel quality of wheat straw would be improved effectively through optimizing the torrefaction conditions. The decomposition of hemicellulose and cellulose was accelerated by the addition of CO_2_ and H_2_O to the reaction atmosphere, leading to a more apparent reduction in the values of H/C and O/C than that of conventional torrefaction performed in an inert atmosphere. The heating value of upgraded wheat straw reached its maximum value after undergoing torrefaction performed in an RFG atmosphere at 573 K. Increasing torrefaction severity could promote the conversion of N-A into N-5. Meanwhile, the free radicals derived from H_2_O can penetrate into the carbon matrix, promoting the decomposition of hydrophilic groups. Additionally, some new reactive oxygen-containing structures would be generated during the oxidative torrefaction process via surface oxidation and gasification. Rising reaction temperature tended to enlarge the specific surface area and pore volume of upgraded wheat straw. With the increase in torrefaction severity, the ignition temperature (602 K → 655 K) and maximum weight loss temperature (697 K → 746 K) for each torrefied sample increased apparently. Otherwise, a rising torrefaction severity tended to decrease the burnout temperature (800 K → 788 K) of the torrefied samples. The upgraded wheat straw obtained from the torrefaction performed in an RFG atmosphere at 573 K expressed the highest reactivity with the lowest reaction activation energy (Ea = 129.30 kJ/mol). Consequently, by means of optimizing torrefaction conditions, the upgradation efficiency and the fuel quality of torrefied samples could be improved significantly. The relevant results of this study will lay the foundation for subsequent investigation on the thermal conversion characteristics of torrefied biomass.

## Figures and Tables

**Table 1:** Basic analysis data of raw and torrefied wheat straw.

Sample Abbreviation	Ultimate Analysis (daf)					Proximate Analysis (db)				HHV (MJ/kg)
	C	H	N	S	O ^a^	M_ar_	Volatile	C_fixed_	Ash	
	(wt%)	(wt%)	(wt%)	(wt%)	(wt%)	(wt%)	(wt%)	(wt%)	(wt%)	
Raw sample	49.44	4.37	1.82	0.51	43.86	7.44	56.13	20.45	15.98	12.88
S1	60.38	5.45	0.84	0.16	33.17	3.09	25.01	41.77	30.13	13.67
S2	63.01	5.32	1.37	0.11	30.19	3.34	20.08	43.01	33.57	13.95
S3	62.66	5.15	0.82	0.08	31.29	3.81	18.58	43.44	34.17	13.78
S4	65.06	4.77	1.34	0.07	28.76	3.35	10.77	49.37	36.51	14.14
S5	65.89	2.87	1.41	0.07	29.76	3.77	9.22	50.37	36.64	14.49
S6	70.98	2.59	1.07	0.05	25.31	3.63	5.54	51.94	38.89	15.26
S7	70.96	1.62	0.93	0.24	26.25	3.37	7.27	51.57	37.79	15.31
S8	72.69	1.01	0.99	0.2	25.11	2.59	4.21	53.98	39.22	16.08
Ash content determination (ad)										
	SiO_2_ (wt.%)	Al_2_O_3_ (wt.%)	Fe_2_O_3_ (wt.%)	CaO (wt.%)	Na_2_O (wt.%)	K_2_O (wt.%)	MgO (wt.%)	Total (wt.%)	Others (wt.%)	
Ash	38.0	7.5	4.7	11.4	5.3	7.1	7.2	81.2	18.8	Ash

^a^ Determined by difference; daf. Dry ash free basis; db. As received basis.

**Table 2:** Kinetic parameters calculated with the FOW method.

Atmosphere	543 K			573 K		
	α	E_a_ (kJ/mol)	R^2^	α	Ea (kJ/mol)	R^2^
Ar	0.1	140.83	0.9943	0.1	167.88	0.9962
	0.2	138.51	0.9975	0.2	164.75	0.9945
	0.3	133.89	0.9997	0.3	178.03	0.9980
	0.4	144.17	0.9995	0.4	194.00	0.9963
	0.5	149.51	0.9999	0.5	185.51	0.9872
	0.6	131.57	0.9953	0.6	159.20	0.9102
	**Average**	**139.75**		**Average**	**174.89**	
6 vol.% O_2_	0.1	89.24	0.9721	0.1	145.58	0.9972
	0.2	124.67	0.9974	0.2	166.71	0.9535
	0.3	145.30	0.9988	0.3	178.48	0.9072
	0.4	158.70	0.9969	0.4	168.64	0.9753
	0.5	152.99	0.9985	0.5	147.60	0.9994
	**Average**	**134.18**		**Average**	**161.40**	
DFG	0.1	89.99	0.9765	0.1	106.43	0.9973
	0.2	128.42	0.9979	0.2	138.56	0.9973
	0.3	154.70	0.9998	0.3	159.55	0.9984
	0.4	171.09	0.9976	0.4	143.13	0.993
	0.5	150.36	0.9879	0.5	107.30	0.9797
	**Average**	**138.91**		**Average**	**130.99**	
RFG	0.1	115.84	0.9999	0.1	99.21	0.9929
	0.2	127.94	0.9998	0.2	128.91	0.9992
	0.3	136.29	0.9982	0.3	145.58	0.9972
	0.4	138.51	0.9975	0.4	146.80	0.9998
	0.5	136.37	0.9908	0.5	125.99	0.9999
	**Average**	**130.99**		**Average**	**129.30**	

**Table 3:** Torrefaction conditions and corresponding abbreviations.

Abbreviation	Temperature	Atmosphere
1	543 K	100 vol.% Ar
2	573 K	100 vol.% Ar
3	543 K	6 vol.% O_2_ balanced Ar
4	573 K	6 vol.% O_2_ balanced Ar
5	543 K	6 vol.% O_2_ + 10 vol.% CO_2_ balanced Ar
6	573 K	6 vol.% O_2_ + 10 vol.% CO_2_ balanced Ar
7	543 K	6 vol.% O_2_ + 10 vol.% CO_2_ + 6 vol.% H_2_O balanced Ar
8	573 K	6 vol.% O_2_ + 10 vol.% CO_2_ + 6 vol.% H_2_O balanced Ar

## Data Availability

The authors do not have permission to share data.

## Abbreviations

The following abbreviations are used in this manuscript:

*A*	Pre-exponential factor (min^−1^)
a	Conversion ratio (%)
C(N)	Nitrogen-containing functional groups
C(O)	Oxygen-containing functional groups
DFG	Dry flue gas
d*a*/d*t*	Conversion rate (%/min)
*E*a	Activation energy (kJ/mol)
*EMCI*	Energy–mass co-benefit index (%)
*f*(a)	Reaction model function
*G*(a)	Integrated form of *f*(a)
HHV	Higher heating value (MJ/kg)
*R*	Universal gas constant (8.314 J mol^−1^ K^−1^)
R^2^	Correlation coefficient
RFG	Raw flue gas
*T*_b_	Burnout temperature (K)
*T*_i_	Ignition temperature (K)
*T*_max_	Maximum weigh loss temperature (K)
*Y*_E_	Energy yield (%)
*Y*_M_	Mass yield (%)
*β*	Heating rate (K/min)
